# Survival among patients cured from gastric adenocarcinoma compared to the background population

**DOI:** 10.1007/s10120-024-01545-y

**Published:** 2024-09-04

**Authors:** Wilhelm Leijonmarck, Fredrik Mattsson, Jesper Lagergren

**Affiliations:** 1 Department of Molecular Medicine and Surgery, Karolinska Institutet and Karolinska University Hospital, Retzius Street 13A, 4th, Floor, 171 77 Stockholm, Sweden; 2 School of Cancer and Pharmacological Sciences, King’s College London, London, UK

**Keywords:** Stomach Neoplasms, Survivors, Cohort Studies, Registers, Sweden / epidemiology

## Abstract

**Background:**

It is unknown if gastric adenocarcinoma survivors have longer, shorter, or similar survival compared to the background population. This knowledge could contribute to evidence-based monitoring strategies, healthcare recommendations, and information for patients and families.

**Methods:**

This population-based cohort study included all patients who underwent gastrectomy for gastric adenocarcinoma between 2006–2015 in Sweden and survived ≥ 5 years after surgery. They were followed up until death, postoperative year 10, or end of study period (31 December, 2020). Division of the observed by the expected survival yielded relative survival rates with 95% confidence intervals (CIs) using the life table method. The expected survival was derived from the entire Swedish population of the corresponding age, sex, and calendar year. Data came from medical records and nationwide registers.

**Results:**

The survival among all 767 gastric adenocarcinoma survivors was shorter than the expected. The reduction in relative survival increased for each follow-up year, from 97.3% (95% CI 95.4–99.1%) year 6 to 86.6% (95% CI 82.3–90.9%) year 10. The decline in relative survival was more pronounced among patients who had gastrectomy in earlier calendar years (82.9% [95% CI 77.4–88.4%] year 10 for years 2011–2015), shorter education (85.2% [95% CI 77.4–93.0%] year 10 for education ≤ 9 years), more comorbidities (78.0% [95% CI 63.9–92.0%] year 10 for Charlson comorbidity score ≥ 2), and no neoadjuvant therapy (83.2% [95% CI 77.4–89.0%] year 10).

**Conclusion:**

Gastric adenocarcinoma survivors seem to have poorer survival than the corresponding background population, particularly in certain subgroups.

## Introduction

Gastric adenocarcinoma (90–95% of all gastric malignancies) carries a poor prognosis with a 5-year overall survival rate of 20–40% [1]. The survival has improved during the last decades, resulting in an increasing number of cancer survivors [1, 2]. Most tumour recurrences occur within 1–3 years after curatively intended gastrectomy and almost all deaths due to recurrences have occurred within 5 years of surgery [3]. Thus, patients who have survived beyond 5 years may be considered cured [3], and routine clinical follow-up is typically discontinued [4, 5]. There is limited evidence regarding how these cancer survivors should be managed by healthcare, advised for the future, and about the expected long-term survival for the patients and their families [6].

This study was initiated after a patient involvement meeting, during which a cancer survivor asked about his long-term survival prospects compared to other people in the population of his age now that he was cured of the cancer. Survivors of gastric adenocarcinoma may have a longer life expectancy because they were selected for surgery due to better general health and fitness [7]. A reduced life expectancy is also possible because some risk factors for gastric adenocarcinoma, e.g., tobacco smoking [8], obesity [9], and dietary factors [10, 11], increase the risk of other lethal conditions, and the cancer treatment could lead to severe sequelae and diseases [12]. One recent study indicated worse survival among gastric cancer survivors than the background population [13], but had some methodological concerns.

With this study, we aimed to help clarify if the survival among survivors of gastric adenocarcinoma is different from the corresponding background population.

## Methods

### Design

This nationwide and population-based cohort study included all patients in Sweden who had undergone gastrectomy for gastric adenocarcinoma from 2006 to 2015 and had survived for at least 5 years after gastrectomy. The patients were followed up for between 6 and 10 postoperative years after the gastrectomy. The observed survival among the gastric adenocarcinoma survivors was compared to the expected survival, which was derived from the entire Swedish background population of the same age, sex, and calendar year. Data came from medical records and nationwide registers. The study was approved by the Regional Ethical Review Board in Stockholm, Sweden (2017/141–31/2).

## Study cohort

The study cohort originated from the Swedish Gastric Cancer Surgery Study (SWEGASS). A detailed description of SWEGASS has been published previously [14]. In brief, SWEGASS includes at least 98% of all patients in Sweden who underwent gastrectomy for gastric adenocarcinoma between 2006 and 2015. Patients were identified in the national *Swedish Cancer Register* [15] and the *Swedish National Patient Register* [16]. These registers have nearly 100% completeness for the recording of gastric adenocarcinoma and gastrectomy [15, 16]. Medical records were reviewed of all patients identified from the registers. Patients who died within 5 years of gastrectomy were excluded.

## Comparison cohort

The comparison cohort comprised the entire Swedish population of the same age, sex, and calendar year as the participants in the study cohort. Data on the entire population were acquired from the *Swedish Register of the Total Population*, which includes all Swedish residents [17].

## Outcome

The study outcome was relative survival. The gastric adenocarcinoma survivors were followed up from the start of year 6 to the end of year 10 after the gastrectomy, until death or end of study period (31 December, 2020), whichever occurred first. Mortality data came from the *Swedish Cause of Death Register*, which is 100% complete for date of death [17, 18].

## Covariates

The following eight covariates, with categorizations in brackets, were included in the analyses: Age (< 66, 66–74, 75–81, or > 81 years at the start of follow-up), sex (male or female), calendar period (2011–2015 or 2016–2020 at the start of follow-up), education level (≤ 9, 10–12, or ≥ 13 years of formal education), comorbidity (0, 1, or ≥ 2 scores according to the Charlson comorbidity index at the date of gastrectomy, not counting the gastric adenocarcinoma), neoadjuvant therapy (no or yes), tumour sub-location in the stomach (cardia or non-cardia), and pathological tumour stage (0-I, II, or III-IV, according to the 8th edition of American Joint Committee on Cancer [AJCC] Cancer Staging Manual [19]). The medical records provided information on age, sex, calendar year, neoadjuvant therapy, tumour sub-location, and pathological tumour stage. Data on years of formal education were retrieved from the *Longitudinal Integrated Database for Health Insurance and Labour Market Studies* (LISA) [20]. Comorbidities were retrieved from the *Swedish National Patient Register* and were classified according to the most well-validated version of the Charlson comorbidity index scoring system [21, 22].

## Statistical analysis

The cumulative relative survival was calculated by dividing the cumulative observed survival in the gastric adenocarcinoma survivor group with the cumulative expected survival, derived from the corresponding background population. The observed survival within the interval *j* $({P}_{j}^{O})$ was calculated by one minus the ratio of the number of cases dying during the interval *j* $({D}_{j})$ to the number of cases alive at the beginning of the interval *j* $({L}_{j})$:${P}_{j}^{O}=1-\frac{{D}_{j}}{{L}_{j}}$

The cumulative observed survival for surviving the interval *x* $({CP}_{X}^{O})$ was obtained by cumulatively multiplying the proportion surviving each interval: ${CP}_{X}^{O}$: ${P}_{1}^{O}*{P}_{2}^{O}*$ …${P}_{x}^{O}$ which was defined as:

$${CP}_{x}^{O}=\prod_{j=1}^{x}{P}_{j}^{O}$$

The expected survival was calculated by matching the gastric adenocarcinoma survivors to the Swedish population of the corresponding age, sex, and calendar year. All matched individuals in the Swedish population were under risk until the corresponding matched patient died or was censored. If ${\widetilde{P}}_{ij}$ is the expected survival of an individual *i* for surviving the interval *j,* then the expected survival for the interval* j* $({P}_{j}^{E})$ was:

$${P}_{j}^{E}=\frac{1}{{L}_{j}}\sum_{i=1}^{{L}_{j}}{\widetilde{P}}_{ij}$$

Cumulative expected survival of surviving interval *x* $(C{P}_{x}^{E})$ was calculated by:

$$C{P}_{x}^{E}=\prod_{j=1}^{x}\left(\frac{1}{{L}_{j}}\sum_{i=1}^{{L}_{j}}{\widetilde{P}}_{ij}\right)$$

For all individuals matched to the patient cohort by age, sex, and calendar year at the beginning the interval *j*, the average of the expected survival in the interval *j* was calculated for *j* = 1,…,*x* and multiplied.

Finally, the relative survival in the interval *j* $({R}_{j})$ was given by:

$${R}_{j}=\frac{{P}_{j}^{O}}{{P}_{j}^{E}}$$

and the cumulative relative survival of surviving the interval *x* $(C{R}_{x})$ was given by:

$$C{R}_{x}=\frac{C{P}_{x}^{O}}{C{P}_{x}^{E}}$$

The observed and expected survival were considered to be statistically significantly different if the confidence interval of the relative survival did not include 100%. Observed and relative survival calculations were also performed for subgroups of the eight covariates, categorized as described above (‘Covariates’). The subgroup analyses were descriptive, thus no formal tests of significance were conducted between groups. In a sensitivity analysis, participants who died due to tumour recurrence of gastric adenocarcinoma were excluded. Because data were 100% complete in the main analysis and missing data were low in the subgroup analyses, we only included cases with complete data in the final analyses, i.e., used the complete case analysis strategy.

A senior biostatistician (FM) was responsible for the data management and statistical analyses. The analyses followed a detailed and pre-defined study protocol and were performed using the statistical software SAS, Version 9.4 (SAS Institute Inc., Cary, NC, USA).

## Results

### Gastric adenocarcinoma survivors

The original SWEGASS cohort included 2154 patients who had undergone gastrectomy for gastric adenocarcinoma between 2006–2015 in Sweden. After exclusion of patients who died within 5 years of gastrectomy (n = 1387), the final study cohort included 767 gastric adenocarcinoma survivors. Characteristics of these survivors are presented in Table 1. Most participants were men, had ≤ 12 years of formal education, no serious comorbidity, no neoadjuvant therapy, non-cardia tumour, and pathological tumour stage 0-I.

**Table 1:** Characteristics of 767 gastric adenocarcinoma survivors who underwent gastrectomy between 2006 and 2015 in Sweden

Characteristic	Number (%)
*Age (in years, at the start of follow-up)*	
< 66	205 (26.7)
66–74	194 (25.3)
75–81	191 (24.9)
> 81	177 (23.1)
*Sex*	
Men	439 (57.2)
Women	328 (42.8)
*Calendar period (at the start of follow-up)*	
2011–2015	388 (50.6)
2016–2020	379 (49.4)
*Years of formal education*	
≤ 9	283 (36.9)
10–12	317 (41.3)
≥ 13	153 (20.0)
Missing	14 (1.8)
*Charlson comorbidity index (at the date of gastrectomy)*	
0	435 (56.7)
1	227 (29.6)
≥ 2	105 (13.7)
*Neoadjuvant therapy*	
No	487 (63.5)
Yes	275 (35.9)
Missing	5 (0.7)
*Tumor sub-location*	
Non-cardia	681 (88.8)
Cardia	82 (10.7)
Missing	4 (0.5)
*Pathological tumor stage*	
0-I	363 (47.3)
II	256 (33.4)
III-IV	132 (17.2)
Missing	16 (2.1)

### Survival in all gastric adenocarcinoma survivors

**Fig. 1:**
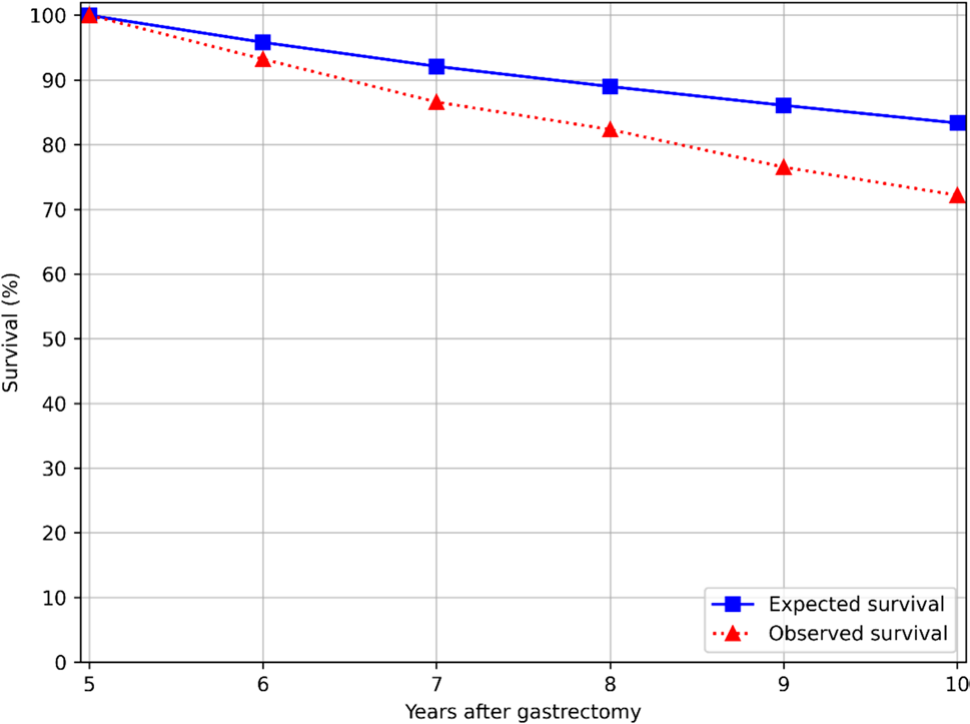
Survival curves for years 5–10 after gastrectomy for gastric adenocarcinoma and expected survival in the entire Swedish population of the same age, sex, and calendar year

The cumulative observed survival among gastric adenocarcinoma survivors gradually decreased from 93.2% (95% CI 91.4–95.0%) at the end of postoperative year 6 to 72.2% (95% CI 68.6–75.8%) at the end of year 10 (Table 2 and Fig. 1). The cumulative relative survival decreased for each year of follow-up, from 97.3% (95% CI 95.4–99.1%) year 6 to 86.6% (95% CI 82.3–90.9%) year 10 (Table 2).

**Table 2:** Observed survival among gastric adenocarcinoma survivors, expected survival in the age, sex, and calendar year-matched background population, and relative survival with 95% confidence intervals (CIs) for the postoperative years 6 to 10

Years after gastrectomy	Alive at the beginning of each year	Observed survival in % (95% CI)	Expected survival in %	Relative survival in % (95% CI)
6	767	93.2 (91.4–95.0)	95.8	97.3 (95.4–99.1)
7	647	86.6 (84.1–89.1)	92.1	94.0 (91.3–96.7)
8	550	82.3 (79.5–85.2)	89.0	92.5 (89.4–95.7)
9	453	76.5 (73.3–79.8)	86.1	88.9 (85.1–92.7)
10	352	72.2 (68.6–75.8)	83.3	86.6 (82.3–90.9)

In the sensitivity analysis excluding participants who died due to tumour recurrence more than 5 years after gastrectomy (n = 34), the cumulative relative survival decreased gradually from 98.7% (95% CI 97.0–100.5%) at the end of year 6 to 90.9% (86.7–95.2%) at the end of year 10 (Supplementary Table 1).

### Survival in subgroups of gastric adenocarcinoma survivors

The relative survival tended to decrease more for gastric adenocarcinoma survivors who underwent gastrectomy during earlier calendar years (82.9% [95% CI 77.4–88.4%] year 10 for the calendar years 2011–2015), had fewer years of formal education (85.2% [95% CI 77.4–93.0%] year 10 for ≤ 9 years of education), had higher Charlson comorbidity index score (78.0% [95% CI 63.9–92.0%] year 10 for scores ≥ 2), and did not receive neoadjuvant therapy (83.2% [95% CI 77.4–89.0%] year 10) (Table 3, 4, 5). There were no major differences in relative survival between subgroups of age, sex, tumour sub-location, or pathological tumour stage (Table 3, 4, 5).

**Table 3:** Observed survival among gastric adenocarcinoma survivors, expected survival in age, sex, and calendar year-matched background population, and relative survival with 95% confidence intervals (CIs) for the postoperative years 6 to 10, stratified by age, sex, and calendar year

Years after gastrectomy	Alive at the beginning of each year	Observed survival in % (95% CI)	Expected survival in %	Relative survival in % (95% CI)
*Age < 66*				
6	205	96.1 (93.5–98.8)	99.5	96.6 (93.9–99.2)
7	178	93.4 (89.9–96.9)	99.0	94.4 (90.9–97.9)
8	161	91.1 (87.0–95.1)	98.4	92.6 (88.5–96.7)
9	138	89.1 (84.5–93.7)	97.7	91.2 (86.5–95.9)
10	114	89.1 (84.5–93.7)	96.9	91.9 (87.2–96.6)
*Age 66–74*				
6	194	95.9 (93.1–98.7)	98.4	97.5 (94.6–100.3)
7	175	91.5 (87.5–95.5)	96.6	94.7 (90.6–98.8)
8	157	86.8 (81.9–91.8)	94.7	91.7 (86.5–96.9)
9	134	83.6 (78.1–89.1)	92.6	90.2 (84.3–96.2)
10	108	79.0 (72.6–85.3)	90.5	87.2 (80.2–94.2)
*Age 75–81*				
6	191	92.7 (89.0–96.4)	96.3	96.2 (92.4–100.0)
7	155	85.5 (80.3–90.7)	92.3	92.7 (87.0–98.3)
8	125	82.1 (76.3–87.9)	87.6	93.7 (87.1–100.2)
9	98	75.4 (68.5–82.3)	83.2	90.6 (82.2–98.9)
10	71	72.2 (64.7–79.7)	77.9	92.7 (83.1–102.4)
*Age > 81*				
6	177	87.6 (82.7–92.4)	88.2	99.3 (93.7–104.8)
7	139	74.3 (67.7–81.0)	77.5	95.9 (87.4–104.5)
8	107	67.4 (60.1–74.7)	68.1	98.9 (88.2–109.6)
9	83	54.4 (46.2–62.6)	59.2	91.9 (78.0–105.7)
10	59	44.3 (35.7–52.9)	50.5	87.6 (70.6–104.6)
*Men*				
6	439	91.6 (89.0–94.2)	95.5	95.9 (93.2–98.6)
7	366	85.8 (82.5–89.2)	91.2	94.1 (90.4–97.7)
8	317	80.7 (76.8–84.5)	87.9	91.8 (87.4–96.2)
9	255	75.0 (70.6–79.4)	84.7	88.6 (83.4–93.7)
10	203	69.8 (65.0–74.7)	81.2	86.0 (80.0–91.9)
*Women*				
6	328	95.4 (93.2–97.7)	96.3	99.1 (96.8–101.5)
7	281	87.6 (83.9–91.3)	93.2	94.0 (90.0–97.9)
8	233	84.6 (80.5–88.7)	90.5	93.5 (88.9–98.0)
9	198	78.6 (73.8–83.5)	87.9	89.5 (83.9–95.0)
10	149	75.5 (70.2–80.8)	86.3	87.5 (81.3–93.6)
*Years 2011–2015*				
6	388	93.6 (91.1–96.0)	96.0	97.5 (95.0–100.1)
7	363	85.8 (82.4–89.3)	92.1	93.2 (89.4–96.9)
8	333	81.2 (77.3–85.1)	88.5	91.7 (87.3–96.1)
9	315	74.0 (69.6–78.3)	85.7	86.3 (81.2–91.4)
10	287	69.3 (64.7–73.9)	83.7	82.9 (77.4–88.4)
*Years 2016–2020*				
6	379	92.9 (90.3–95.5)	95.7	97.1 (94.4–99.8)
7	284	87.6 (84.2–91.1)	92.1	95.2 (91.4–98.9)
8	217	84.0 (79.9–88.1)	89.7	93.7 (89.1–98.2)
9	138	81.6 (77.0–86.2)	87.0	93.8 (88.5–99.1)
10	65	79.1 (73.4–84.7)	81.9	96.5 (89.6–103.3)

**Table 4:** Observed survival among gastric adenocarcinoma survivors, expected survival in the age, sex, and calendar year-matched background population, and relative survival with 95% confidence intervals (CIs) for the postoperative years 6 to 10, stratified by education level and comorbidity

Years after gastrectomy	Alive at the beginning of each year	Observed survival in % (95% CI)	Expected survival in %	Relative survival in % (95% CI)
* ≤ 9 years of formal education*				
6	283	91.5 (88.3–94.8)	94.5	96.8 (93.4–100.2)
7	235	85.3 (81.1–89.5)	90.3	94.4 (89.7–99.1)
8	205	82.0 (77.3–86.6)	87.0	94.2 (88.9–99.6)
9	174	75.4 (70.0–80.8)	83.2	90.6 (84.1–97.1)
10	136	67.6 (61.4–73.8)	79.4	85.2 (77.4–93.0)
*10–12 years of formal education*				
6	317	93.1 (90.3–95.9)	96.3	96.6 (93.7–99.5)
7	277	85.7 (81.7–89.6)	92.6	92.5 (88.2–96.7)
8	232	81.6 (77.2–86.0)	89.3	91.4 (86.5–96.4)
9	197	75.4 (70.3–80.5)	87.0	86.6 (80.8–92.5)
10	151	72.9 (67.5–78.3)	84.4	86.3 (80.0–92.7)
* ≥ 13 years of formal education*				
6	153	98.0 (95.8–100.2)	97.2	100.8 (98.6–103.1)
7	125	92.6 (88.1–97.0)	94.3	98.1 (93.4–102.8)
8	106	85.6 (79.3–91.8)	92.5	92.5 (85.7–99.2)
9	77	83.3 (76.6–90.1)	90.7	91.9 (84.4–99.4)
10	61	82.0 (74.8–89.2)	89.4	91.7 (83.7–99.8)
*Charlson comorbidity index 0*				
6	435	94.9 (92.9–97.0)	96.2	98.7 (96.5–100.8)
7	369	87.7 (84.5–90.9)	92.5	94.9 (91.4–98.4)
8	317	84.4 (80.8–88.0)	89.6	94.2 (90.2–98.2)
9	276	79.5 (75.4–83.6)	86.8	91.6 (86.9–96.4)
10	215	75.5 (70.9–80.0)	84.0	89.8 (84.4–95.2)
*Charlson comorbidity index 1*				
6	227	93.0 (89.6–96.3)	95.9	97.0 (93.5–100.4)
7	197	86.4 (81.8–90.9)	92.3	93.6 (88.7–98.5)
8	162	81.6 (76.3–86.8)	89.5	91.1 (85.2–97.0)
9	124	75.0 (68.8–81.2)	86.4	86.8 (79.6–94.0)
10	96	71.1 (64.3–77.8)	84.3	84.3 (76.3–92.4)
*Charlson comorbidity index ≥ 2*				
6	105	86.7 (80.2–93.2)	94.0	92.2 (85.3–99.1)
7	81	82.4 (75.0–89.8)	90.1	91.4 (83.2–99.6)
8	71	75.4 (66.8–84.1)	84.9	88.9 (78.7–99.0)
9	53	66.9 (56.9–76.9)	81.8	81.8 (69.6–94.0)
10	41	60.4 (49.5–71.2)	77.4	78.0 (63.9–92.0)

**Table 5:** Observed survival among gastric adenocarcinoma survivors, expected survival in the age, sex, and calendar year-matched background population, and relative survival with 95% confidence intervals (CIs) for the postoperative years 6 to 10, stratified by neoadjuvant therapy, tumour sub-location, and pathological tumour stage

Years after gastrectomy	Alive at the beginning of each year	Observed survival in % (95% CI)	Expected survival in %	Relative survival in % (95% CI)
*No neoadjuvant therapy*				
6	487	92.8 (90.5–95.1)	94.5	98.3 (95.8–100.7)
7	419	84.2 (80.9–87.5)	89.8	93.7 (90.0–97.4)
8	353	78.5 (74.7–82.3)	86.0	91.3 (86.8–95.7)
9	299	71.1 (66.8–75.4)	82.6	86.1 (80.9–91.3)
10	235	66.3 (61.6–70.9)	79.7	83.2 (77.4–89.0)
*Neoadjuvant therapy*				
6	275	93.8 (91.0–96.7)	98.2	95.5 (92.6–98.4)
7	223	91.3 (87.9–94.7)	96.3	94.8 (91.3–98.3)
8	193	89.9 (86.2–93.6)	94.5	95.1 (91.2–99.1)
9	151	87.5 (83.2–91.8)	92.9	94.2 (89.6–98.8)
10	114	84.4 (79.3–89.5)	90.8	93.0 (87.4–98.6)
*Non-cardia location*				
6	681	93.3 (91.4–95.1)	95.7	97.5 (95.5–99.4)
7	577	86.5 (83.8–89.1)	91.8	94.2 (91.3–97.0)
8	492	81.9 (78.9–84.9)	88.7	92.4 (88.9–95.8)
9	403	76.4 (72.9–79.9)	85.8	89.1 (85.0–93.1)
10	311	72.0 (68.2–75.8)	83.0	86.7 (82.1–91.3)
*Cardia location*				
6	82	92.7 (87.1–98.3)	97.1	95.5 (89.7–101.3)
7	66	87.1 (79.6–94.6)	94.7	91.9 (84.0–99.9)
8	54	85.5 (77.4–93.5)	92.1	92.8 (84.1–101.5)
9	47	76.4 (66.0–86.8)	89.0	85.8 (74.1–97.5)
10	38	72.3 (61.1–83.6)	86.9	83.2 (70.3–96.1)
*Pathological tumor stage 0-I*				
6	363	93.9 (91.5–96.4)	96.4	97.5 (94.9–100.0)
7	310	90.0 (86.9–93.2)	92.9	96.9 (93.5–100.2)
8	276	85.8 (82.0–89.5)	89.8	95.5 (91.3–99.6)
9	230	80.5 (76.1–85.0)	87.3	92.3 (87.2–97.3)
10	187	75.8 (70.8–80.8)	84.5	89.7 (83.8–95.5)
*Pathological tumor stage II*				
6	256	93.0 (89.8–96.1)	95.1	97.8 (94.5–101.1)
7	213	82.5 (77.7–87.3)	91.0	90.7 (85.4–96.0)
8	176	79.2 (74.0–84.4)	87.9	90.1 (84.2–96.1)
9	145	71.0 (64.9–77.1)	84.4	84.1 (76.9–91.3)
10	105	67.0 (60.4–73.5)	81.7	82.0 (74.0–90.0)
*Pathological tumor stage III-IV*				
6	132	90.9 (86.0–95.8)	96.0	94.7 (89.6–99.8)
7	109	83.4 (76.9–89.9)	92.7	90.0 (83.0–97.0)
8	83	78.4 (70.9–85.8)	89.9	87.2 (78.9–95.5)
9	65	74.8 (66.6–82.9)	87.3	85.7 (76.3–95.0)
10	49	71.7 (62.9–80.6)	85.6	83.8 (73.5–94.2)

## Discussion

This study found that survivors of gastric adenocarcinoma have gradually poorer survival compared to the corresponding background population between 6 and 10 years after gastrectomy. The decreased relative survival was seemingly more pronounced in earlier calendar years and in participants with fewer years of education, more comorbidities, and without neoadjuvant therapy, whereas there were no obvious differences comparing subgroup of age, sex, tumour sub-location, and pathological tumour stage.

Methodological strengths of this study include the nationwide and population-based design with a high participation rate, which provided a large and unselected cohort and generalizable results. The review of all medical records and the use of high-quality nationwide registers provided accurate and nearly complete information on all variables used in the study. This study also has limitations. There was a limited number of patients remaining many years after gastrectomy. Therefore, we restricted the number of follow-up years to 10 to secure statistical power. We did not have health data on the background Swedish population, which was used as the comparison group, and thus could not assess mechanisms for the differences in survival.

One recent study based on data from the database Surveillance, Epidemiology, and End Results (SEER) in the United States observed an increased mortality among 5-year gastric cancer survivors compared to the background population (standardized mortality ratio 1.72, 95% CI 1.66–1.77) [13]. However, that study included all types of gastric malignancies with different treatments and prognoses, did not account for late deaths due to tumour recurrence, and the study period started already in 1975 raising concerns about its relevance for healthcare of today. Nevertheless, the finding is consistent with the results of the present study. Other studies of earlier cohorts of gastric cancer survivors diagnosed many decades ago have found a relative survival around 80–90% in Western cohorts [23–25], i.e. consistent with the present study, and up to 97% in Japan [26, 27]. A similarly designed study on oesophageal cancer survivors in Sweden (from our group) revealed a pattern of decreased survival compared to the corresponding background population [28]. Given the anatomical proximity of the two cancer types, several shared risk factors, and similar treatment, particularly for adenocarcinomas of the gastroesophageal junction, the similar results of the present study indicate consistency.

The current study revealed that not only the entire group of gastric adenocarcinoma survivors had a decreased survival compared to the expected, but all subgroups showed a tendency of poorer survival compared to the corresponding background population, including survivors with characteristics typically associated with better long-term survival. This observation suggests that other factors play a major role. Risk factors for gastric adenocarcinoma, such as tobacco smoking, obesity, and dietary factors [10, 29], are shared with other serious conditions, and long-term consequences could arise from the surgical procedure or chemotherapy [12]. This could contribute to a higher prevalence of severe comorbidities compared to the background population. Previous studies have indicated an elevated risk of several conditions in gastric adenocarcinoma survivors when compared to the general population, including osteoporosis [30], anaemia [31], Alzheimer’s disease [32], and second primary cancers [33, 34]. Some specific causes of death are also more common among gastric cancer survivors than the general population, particularly infectious diseases, chronic liver disease, other malignancies, and renal diseases [13]. Taken together, there are several explanations for the worse survival rates found among patients having been cured of gastric adenocarcinoma compared to the background population in the present study.

The analyses indicated worse survival in some subgroups of participants. The worse survival among patients who underwent gastrectomy during earlier calendar years may be due to recent developments in diagnostic procedures and patient assessment, rendering a stricter selection of patients for surgery [35]. The lower survival in gastric adenocarcinoma survivors with less education might be due to lifestyle factors, e.g., higher exposure to tobacco smoking, overconsumption of alcohol, and obesity [8–11]. Among all subgroups analysed, the relative survival was worst among those with multiple comorbidities (Charlson comorbidity index ≥ 2), suggesting that this is the strongest risk factor for mortality among gastric adenocarcinoma survivors. Finally, there were lower relative survival rates among patients who did not receive neoadjuvant therapy compared to those who did. Patients who did not receive neoadjuvant therapy might have poorer general health and a thus lower chance of long-term survival, but research is warranted to identify the mechanisms behind this less expected observation.

In conclusion, this nationwide and population-based study found that gastric adenocarcinoma survivors have a gradually worse survival 6–10 years after gastrectomy compared to the corresponding background population, and even more so in certain subgroups. This finding underscores the relevance of closer monitoring and health-related recommendations for these individuals and provides evidence-based information regarding the expected long-term survival to these individuals and their families.

## Data Availability

Data not available.
